# Apigenin 7-glucoside impedes hypoxia-induced malignant phenotypes of cervical cancer cells in a p16-dependent manner

**DOI:** 10.1515/biol-2022-0819

**Published:** 2024-03-20

**Authors:** Yan Li, Xiaoli Man, Qing Zhang, Xiaowu Wang, Yongli Yang

**Affiliations:** Department of Gynecologic Oncology, The Affiliated Hospital of Qinghai University & Affiliated Cancer Hospital of Qinghai University, Xining, China; Department of Gynecology, Puyang Maternity and Child Care Centers, Puyang, China; Department of Gynecology and Obstetrics, The People’s Hospital of Luyi, Luyi, China; Department of Surgical Oncology, The Affiliated Hospital of Qinghai University, No. 29, Tongren Road, Xining, 810000, China; Department of Gynecology, The Affiliated Hospital of Qinghai University & Affiliated Cancer Hospital of Qinghai University, No. 29, Tongren Road, Xining, 810000, China

**Keywords:** hypoxia, apigenin 7-glucoside, cervical cancer, p16

## Abstract

Apigenin 7-glucoside (A7G) can suppress cell proliferation and trigger apoptosis in cervical cancer cells. Considering that hypoxia is associated with the malignant phenotypes in cervical cancer, this study aimed to uncover whether A7G exhibits suppressive effects on the hypoxia-induced malignant phenotype of cervical cancer cells (HeLa cells). Compared to normoxia, hypoxia can enhance the malignant phenotypes of HeLa cells, including cell proliferation, reduced sensitivity against chemotherapeutic agents (oxaliplatin and paclitaxel), cancer stemness, migration, and invasion. A7G intervention (20, 40, and 60 μM) could impair these malignant phenotypes of HeLa cells and upregulate the expression level of total and nuclear p16 proteins. Molecular docking analysis showed the interaction between anion exchanger 1 and A7G. In p16-silencing HeLa cells, the anticancer effects of A7G were absent. Therefore, hypoxia derives malignant phenotypes of HeLa cells, which could be impeded by A7G in a p16-dependent manner.

## Graphical abstract

**Figure:**
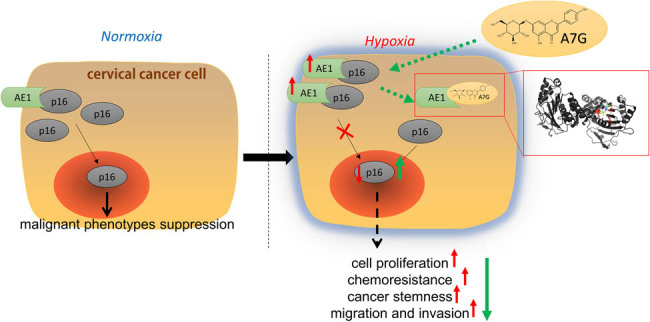

## 1 Introduction

As the fourth most common female malignancy, cervical cancer accounted for more than 300,000 deaths in 2020 worldwide [1]. Considering limited treatment options and economic and cultural factors, a vast difference (approximately 18-fold) in the mortality rate of cervical cancer is observed between low-income developing countries and developed countries [2]. In the case of surgical resection, local recurrence occurs in more than 30% of patients with locally advanced-stage cervical cancer [3]. To date, chemotherapy and radiotherapy have become the standard therapeutic strategies for patients with locally advanced-stage cervical cancer [4]. Despite recent advancements in radiotherapy and chemotherapy, local cervical cancer with advanced stage is likely to display resistance, which contributes to poor prognosis [5]. Hence, uncovering the mechanism underlying the development of malignant phenotypes in cervical cancer is important to address the radio- or chemo-resistance in cervical cancer treatment.

Exposing cells to severe conditions, such as hypoxia, can cause alterations in the genome, which may strengthen the malignant potential of tumor cells and resistance to anticancer treatment [6]. As a hallmark of solid tumor microenvironment, hypoxia helps cancer cells respond adaptively to meet the needs of carcinogenesis, cancer progression, and relapse. Hypoxia may cause several conditions, such as increasing spontaneous damage to DNA or DNA repair suppression and regulating p53 expression and angiogenesis, to help cancer cells respond adaptively to meet the needs of cancer progression and relapse [7,8,9]. Hence, the existence of hypoxic cells contributes to resistance against chemotherapy and local failure [10,11]. In the past two decades, hypoxic cells in tumors were directly measured by using special techniques, which verified the existence of hypoxic tumor cells within human cancers [12]. Moreover, increasing evidence suggests that tumor hypoxia is closely related to prognosis in several cancers, including cervical, breast, and other solid malignancies [13]. A recent study has proven that hypoxic cervical cancer cells had a survival advantage compared with the normixic one and revealed that hypoxia exposure was critical for the development of radio resistance [14]. In addition, hypoxia is negatively correlated with the response to treatment and survival rates in patients with cervical cancer [15,16]. Therefore, hypoxia confers cervical tumor’s stronger abilities of relapse and death as well as resistance to chemotherapy [17]. Therefore, to treat cervical cancer, a promising approach would be to manipulate hypoxic stress.

Natural products are considered potential alternatives for chemotherapy drugs, which can be used in combination with chemotherapy agents because of their diverse biological activities and wide range of sources [18]. Despite tremendous efforts in preclinical studies to develop natural products targeting hypoxia-inducible factor-1 over the past decade, the efforts to produce clinically available treatments have been unsuccessfully [19]. As a natural compound widely distributed in most plants, apigenin 7-glucoside (A7G) has been proven to exert a therapeutic potential on multiple pathological disorders, such as cognitive impairment, oxidative stress, inflammation, and cancer progression [20,21,22]. For example, A7G presents an anti-inflammatory effect with a safety property by suppressing the secretion of TNF-α cytokine in lipopolysaccharide-induced macrophages [20]. A7G not only suppressed HL-60 cell growth in a dose- and time-dependent manner but also stimulated granulocytic differentiation of HL-60 cells [22]. Moreover, a recent study has highlighted the antitumor effect of A7G on cervical cancer, as revealed by the cell apoptosis and impaired cell migration of HeLa cells after A7G intervention [23]. However, the effects of A7G on hypoxia-induced malignant phenotypes of human cervical cancer cells, as well as the underlying mechanisms, remain unclear. Hence, the present study aimed to explore the effects of A7G on the biological characteristics of cervical cancer cells under hypoxia and provide insights into the strategies for overcoming resistance in cervical cancer.

In this study, we found that malignant phenotypes, including stronger proliferative ability, chemo-resistance, cancer stemness, migration, and invasion, were enhanced in cervical cancer HeLa cells after exposure to hypoxia. Treatment with A7G hindered malignant phenotypes of HeLa cells under hypoxia and regulated the p16 signaling *in vitro*. Our findings may provide comprehensive understanding of the potential anticancer properties of A7G on cervical cancer.

## 2 Materials and methods

### 2.1 Cell culture and treatment

Human cervical cancer cell line HeLa was obtained from ATCC (VA, USA) and was grown in DMEM medium (Gibco; Thermo Fisher Scientific, IL, USA) containing 1% penicillin–streptomycin (Gibco) and 10% FBS (Gibco). Hela cells were cultivated in a humidified environment of 5% CO_2_ at 37°C. The concentrations (20, 40, and 60 μM) of A7G used in this study were selected in accordance with a previous study [23]. In mimicking the tumor microenvironment with low oxygen, the incubator was adjusted to a saturated humidity of 37°C, with 5% CO_2_, 94% N_2_, and 1% O_2_, for hypoxic condition culture.

### 2.2 Colony formation

Approximately 500 HeLa cells received different transfection and treatments and seeded into six-well plates (Corning, NY, USA). After cultivation in a 5% CO_2_ incubator under hypoxic and normoxic conditions at 37°C for 2 weeks, cell colonies were fixed with 4% paraformaldehyde before 10 min staining of 1% crystal violet (Sigma–Aldrich, Germany). The dishes were gently washed, counted, and photographed under a BX51 microscope (Olympus, Tokyo, Japan).

### 2.3 Cell counting kit-8 assay

Cell counting kit-8 (CCK-8) assay was conducted to evaluate the resistance ability against oxaliplatin or paclitaxel of HeLa cells from different groups. In brief, HeLa cells with diverse interventions were grown in 96-well plates and subsequently treated with a series of oxaliplatin concentrations (5, 10, 20, and 40 μM) or paclitaxel (2.5, 5, 10, and 20 μM) under hypoxic or normoxic conditions for 24 h. Afterward, 10% of CCK-8 solution (Dojindo Laboratories, Kumamoto, Japan) was added for additional 1.5 h incubation. Finally, the absorbance (450 nm) was measured under a microplate reader (Bio-Rad, CA, USA) to calculate the IC_50_ value.

### 2.4 Western blot analysis

After isolating the total protein from HeLa cells with RIPA buffer (10×, Cell Signaling, MA, USA), protein quantification was performed using a BCA assay kit (Sigma–Aldrich). Meanwhile, the nuclear extraction kit (Thermo Scientific, MA, USA) was applied to purify the nuclear proteins. The protein was separated via sodium dodecyl-sulfate polyacrylamide gel electrophoresis gel and subsequently transferred onto polyvinylidene fluoride membranes. After blocking with 5% skim milk, membranes were incubated with primary antibodies specific for SOX2 (Invitrogen, MA, USA; #14-9811-82; 1 µg/mL), ALDH1 (Abcam, MA, USA; #ab9883; 0.5 µg/mL), Nanog (Invitrogen; #14-5,761-80; 2 µg/mL), p16 (Cell Signaling; #80772; 1:1,000), AE1 (Abcam; #ab9286; 1 µg/mL), Lamin A (Abcam; #ab108595; 1:12,000), and GAPDH (Beyotime, Jiangsu, China; #AF5009; 1:1,000) overnight. Next, membranes were washed three times before incubation with an horseradish peroxidase-conjugated secondary antibody (Beyotime, #A0208; 1:1,000) for 2 h. Finally, an enhanced chemiluminescence detection reagent (Sigma–Aldrich) was used to visualize protein bands. Densitometric analysis with ImageJ software was performed, and Lamin A and GAPDH were used as loading control for protein from nuclear and whole cell, respectively.

### 2.5 Wound healing assay

After the intervention, 8 × 10^4^ HeLa cells were seeded onto 6-well plates and cultivated. After forming a monolayer of cells, scratches of similar size were created in the cell layer of each well. Then, scratched cells were removed, and the remaining cells were treated with different agents and subjected to hypoxic or normoxic conditions for another 24 h cultivation. The same position of scratches was photographed by using a BX51 microscope at 0 and 24 h after scratching.

### 2.6 Transwell assay

The invasion of HeLa cells was evaluated by using Transwell chambers (Corning). In brief, cells with or without transfection were resuspended in 200 μL of RPMI 1640 with diverse agents and added into the upper chambers. Meanwhile, 700 μL of RPMI 1640 with serum was added to the lower chamber. The Transwell chambers were subjected to 24 h incubation under hypoxic or normoxic conditions. Afterward, the cells in the upper chamber were wiped, whereas cells traversing the membranes to the lower chamber were subjected to fixation with 4% paraformaldehyde prior to staining with 0.1% crystal violet (Sigma–Aldrich). Finally, under a BX51 microscope, the stained HCT116 cells were imaged and counted (five random visual fields).

### 2.7 Cell transfections

HeLa cells divided into the diverse groups were subjected to different treatments: scrambled negative control small-interfering RNA (si-NC) group (cells transfected with irrelevant nucleotides to act as a negative control), small-interfering RNA (siRNA) that specifically targeted the p16 (si-p16)#1 group (cells transfected with a siRNA#1 that specifically targeted p16), si-p16#2 group (cells transfected with siRNA#2 that specifically targeted p16), si-p16#3 group (cells transfected with siRNA#3 that specifically targeted p16), si-NC + A7G group (cells transfected with irrelevant nucleotides prior to treatment with 60 μM A7G), si-p16 group (cells transfected with si-p16#3 prior to treatment with 0.05% DMSO), and si-p16 + A7G group (cells transfected with si-p16#3 prior to treatment with 60 μM A7G). Cell transfection was conducted for 48 h by using a Lipofectamine^®^ 3000 reagent (Invitrogen). The efficiency of all transfections was examined by detecting protein levels using Western blot.

### 2.8 Molecular docking analysis

The crystal structure of the regulatory domain of AE1 and the 3D structure of A7G were obtained from the Protein Data Bank (ID: 5JHO) and PubChem chemical library (ID: 5280704), respectively. Autodock tools were utilized for docking simulation of A7G and predicting its binding affinity with the AE1. Finally, the PyMol tool (version 1.3, Schrödinger, LLC, New York, USA) was applied to visualize the results.

### 2.9 Statistical analysis

All data were expressed as mean ± standard error of the mean. The Student’s *t*-test and one-way analysis of variance followed by Bonferroni’s method were, respectively, applied for comparison between two-group and multiple-group conditions based on GraphPad Prism version 8.0.1 (GraphPad Software, CA, USA). *P* values <0.05 were considered statistically significant.

## 3 Results

### 3.1 Malignant behaviors of cervical cancer cells were aggravated under hypoxic conditions

**Figure 1:**
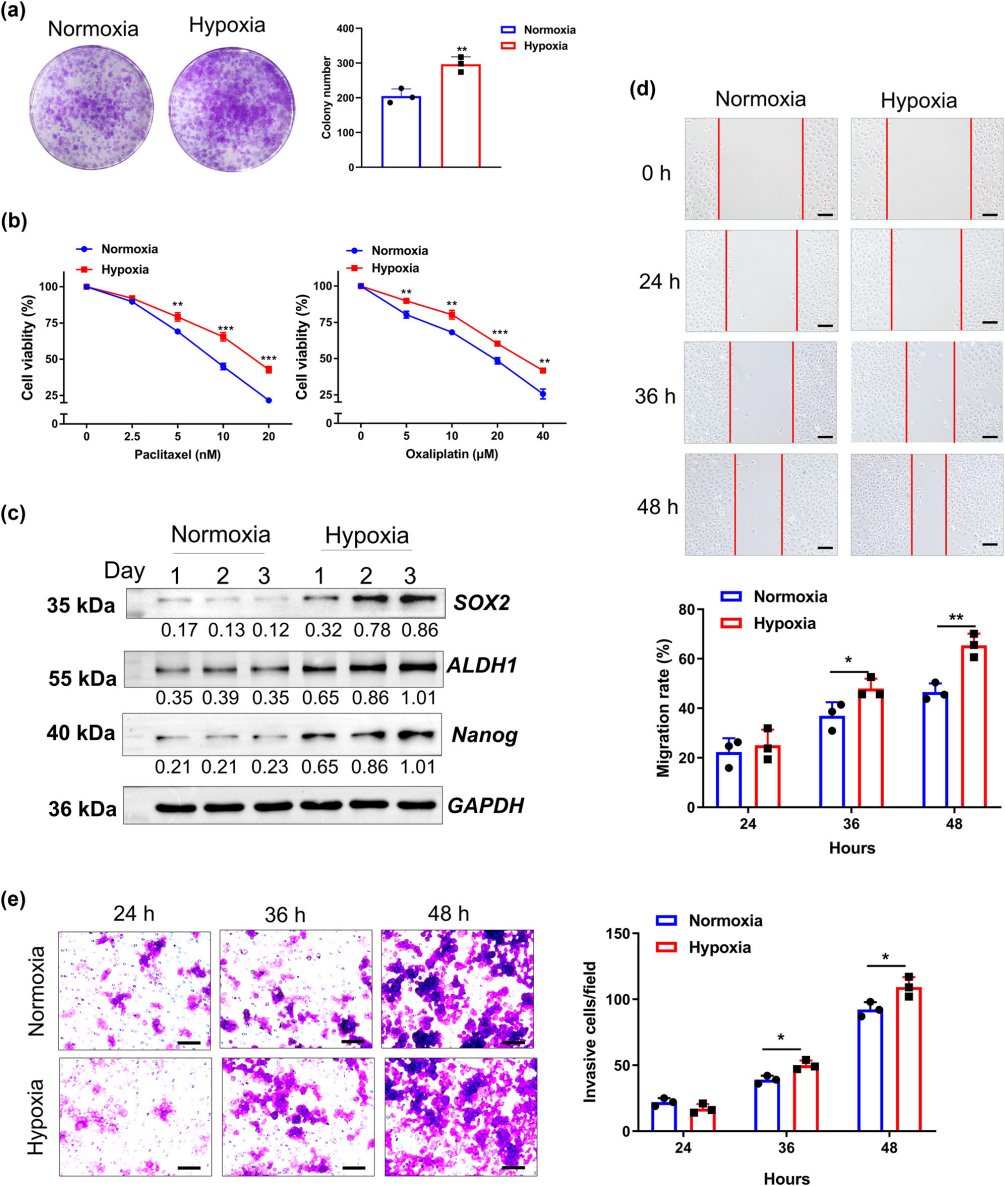
Malignant behaviors of cervical cancer cells were aggravated under hypoxic conditions. The Hela cells were cultured under normoxic (21% O_2_) and hypoxic (1% O_2_) conditions. (a) Colony formation assay assessed the proliferative ability of HeLa cells. (b) CCK-8 assay assessed the ability of HeLa cells to resist oxaliplatin and paclitaxel. (c) Western blotting assessment of the protein level of stemness-related proteins (SOX2, ALDH1, and Nanog). (d) Wound healing assay assessed the migration of HeLa cells. (e) Transwell assay assessed the invasive ability of HeLa cells. *n* = 3. Data are presented as the means ± SEM. *P* value was based on Student’s *t*-test. *P < 0.05, **P < 0.01, ***P < 0.001 versus the normoxia group.

The proliferation, resistance to chemotherapy, stemness, migration, and invasion of HeLa cells were assessed under different oxygen concentrations to investigate whether hypoxia can strengthen the malignant phenotypes of cervical cancer cells. Under a hypoxic environment, HeLa cells acquired more aggressive phenotypes (Figure 1a–e). Colony formation assay showed that the HeLa cells cultured under hypoxia formed more number of colonies than those under normoxia (Figure 1a). Moreover, hypoxia confers the resistance of HeLa cells against chemotherapeutic agents (oxaliplatin and paclitaxel, Figure 1b). The expression level of stemness-related proteins (SOX2, ALDH1, and Nanog) in HeLa cells under hypoxia was significantly higher than those under normoxia (Figure 1c). Similarly, compared with the normoxia environment, hypoxia significantly enhanced the migrative and invasive abilities of HeLa cells (Figure 1d and e).

### 3.2 A7G effectively impeded hypoxia-induced malignant phenotypes of cervical cancer cells

**Figure 2:**
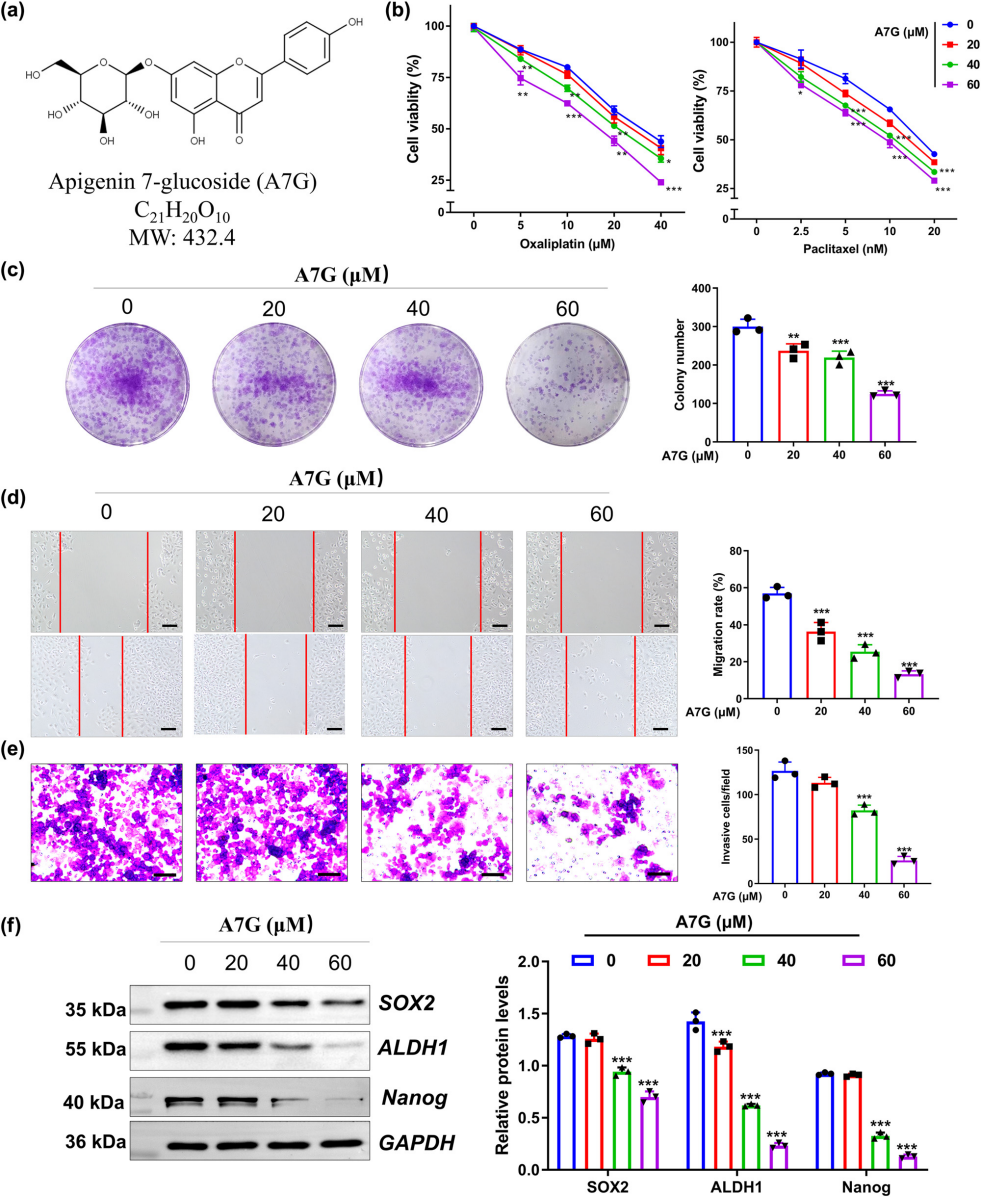
A7G effectively impeded the hypoxia-induced malignant phenotypes of cervical cancer cells. Under hypoxic conditions, Hela cells were subjected to different doses of A7G (0, 20, 40, and 60 μM) before a series of biological function experiments. (a) Chemical structure formula of A7G. (b) CCK-8 assay assessed the ability of HeLa cells to resist oxaliplatin and paclitaxel. (c) Colony formation assay assessed the proliferative ability of HeLa cells. (d) Wound healing assay assessed the migration of HeLa cells. (e) Transwell assay assessed the invasive ability of HeLa cells. (f) Western blotting assessment of the protein level of stemness-related proteins (SOX2, ALDH1, and Nanog). *n* = 3. Data are presented as the means  ±  SEM. *P* value was based on one-way analysis of variance. *P < 0.05, **P < 0.01, ***P < 0.001 versus the 0 μM group.

Although a recent study has indicated the antitumor effect of A7G (Figure 2a) on cervical cancer, we wonder whether A7G intervention can influence the hypoxia-induced malignant phenotypes of cervical cancer cells. Under hypoxic conditions, HeLa cells were subjected to different doses of A7G (0, 20, 40, and 60 μM) before a series of biological function experiments. As shown in Figure 2a–f, the low dose of A7G (20 μM) has little influence on the resistance of HeLa cells against chemotherapy, proliferation, stemness, migration, and invasion under hypoxic conditions. In addition, 40 and 60 μM of A7G evidently enhanced the sensitivity of HeLa cells to oxaliplatin and paclitaxel (Figure 2b). Colony formation, wound healing, and Transwell assays also suggested that A7G significantly impeded the proliferation, migration, and invasion of HeLa cells in a dose-dependent manner (Figure 2c–e). Similarly, for the detection of stemness-related proteins, A7G intervention led to the downregulation of SOX2, ALDH1, and Nanog in HeLa cells in a dose-dependent manner (Figure 2f).

### 3.3 A7G promoted the activation of p16 by interacting with AE1 in cervical cancer cells

**Figure 3:**
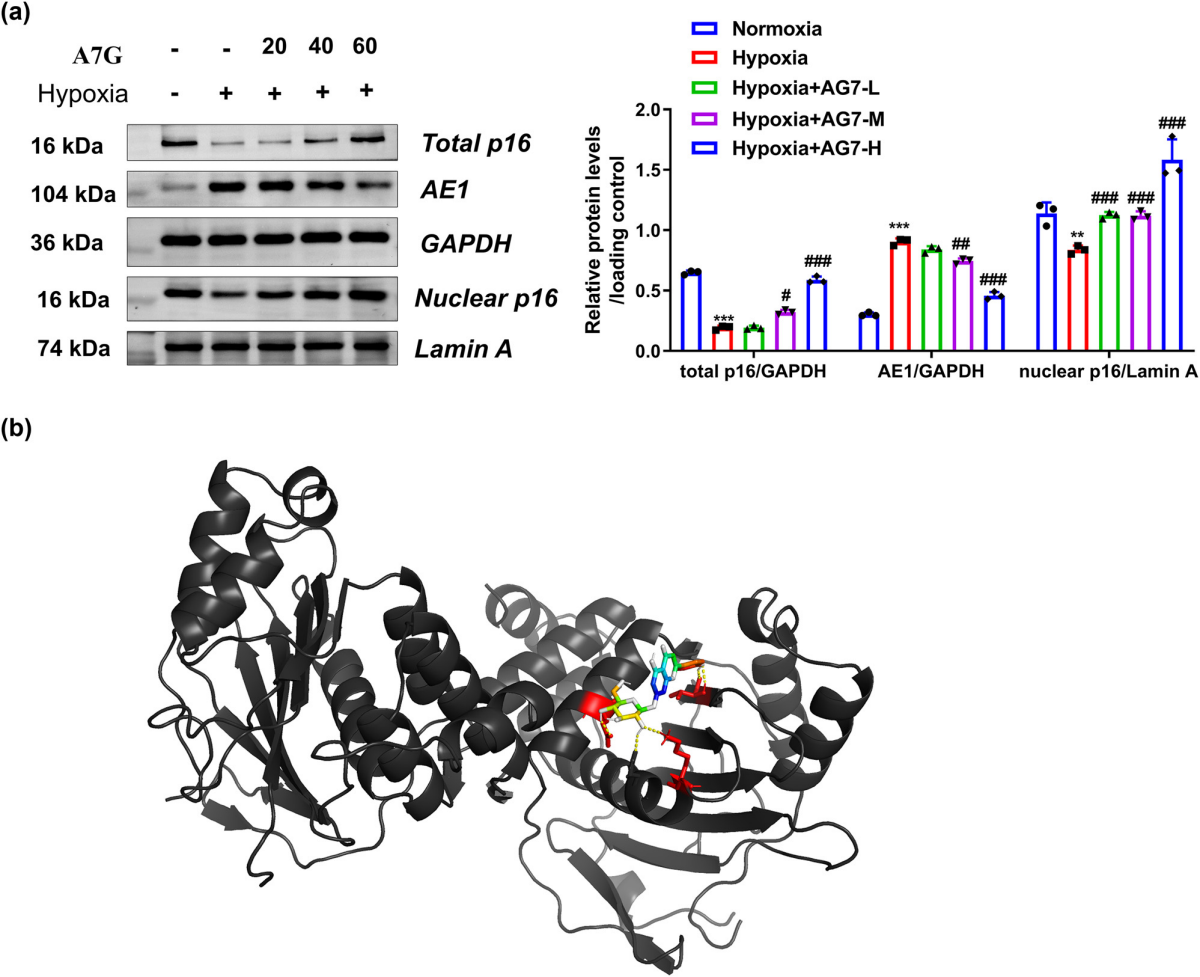
A7G promoted the activation of p16 by interacting with AE1 in hypoxic cervical cancer cells. (a) Western blotting assessment of AE1, total p16, and nuclear p16 protein levels in HeLa cells under normoxic conditions or intervened with different doses of A7G (0, 20, 40, and 60 μM) under hypoxic conditions. (b) Molecular docking diagram of A7G with AE1. *n* = 3. Data are presented as the means ± SEM. *P* value was based on one-way analysis of variance. ***P < 0.001 versus the normoxia group; ^#^ *P* < 0.05, ^##^ *P* < 0.01, ^###^ *P* < 0.001 versus the hypoxia group.

Compared with normoxia, hypoxia significantly downregulated total and nuclear p16 protein levels and remarkably upregulated the AE1 protein level of HeLa cells (Figure 3a). Moreover, A7G intervention can reverse the changes induced by hypoxia in a dose-dependent manner (Figure 3a). In a previous report, a direct interaction was observed between p16 and AE1 and that the abundant expression of AE1 protein is closely related to cancer progression via p16 cytoplasmic sequestration [24]. Then, molecular docking analysis revealed a potential interaction between A7G with AE1 (Figure 3b). These results indicated that A7G could enhance the activation of p16 by interacting with AE1.

### 3.4 Knockdown of p16 blocked the effect of A7G on the hypoxia-induced malignant phenotypes of cervical cancer cells

**Figure 4:**
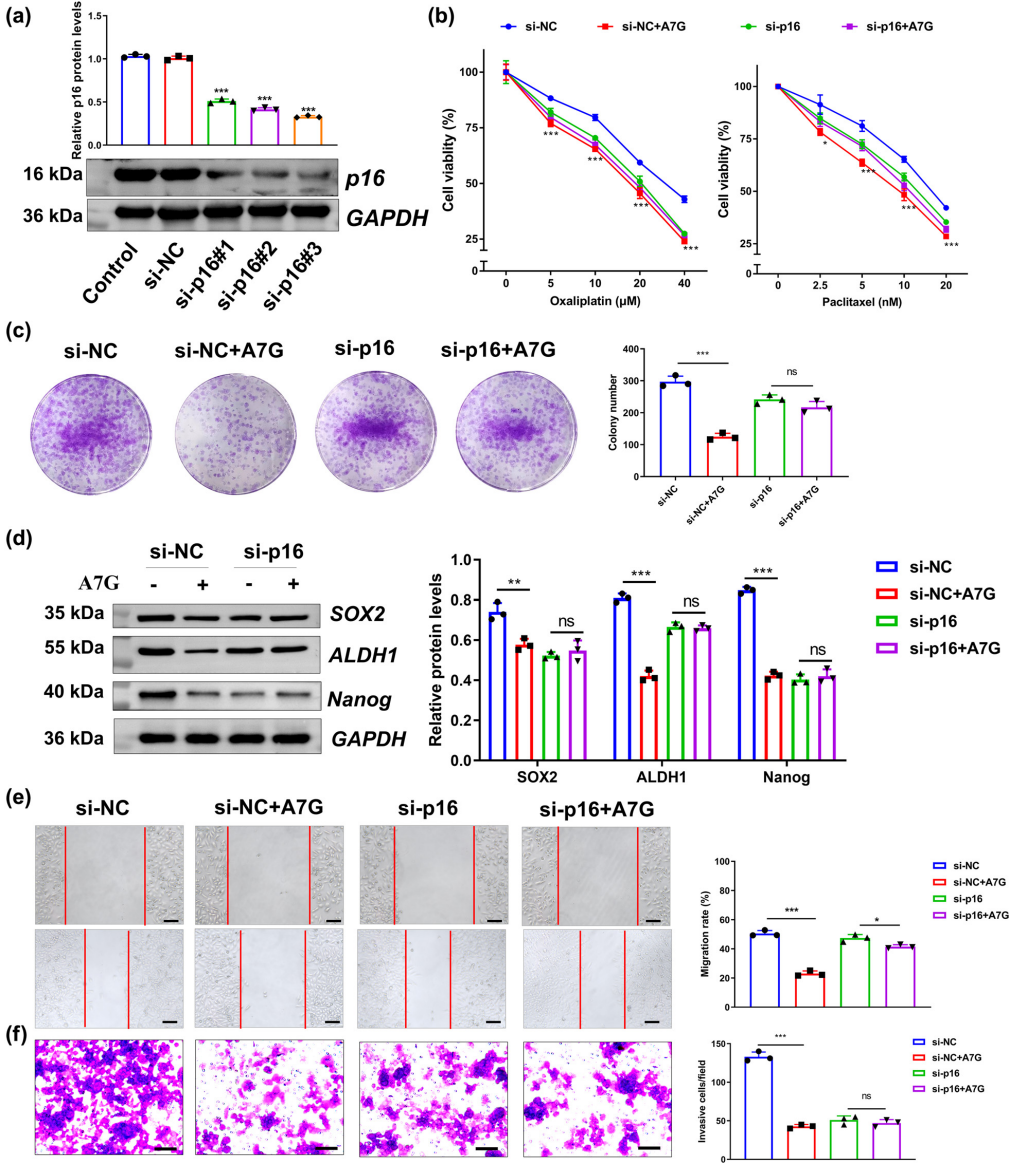
Knockdown of p16 blocked the effect of A7G on the hypoxia-induced malignant phenotypes of cervical cancer cells. (a) Western blotting assessment of the protein level of p16 in HeLa cells transfected with si-NC, si-p16#1, si-p16#2, or si-p16#3 under hypoxic conditions. The si-p16#3 was selected for further study as its knockdown efficiency was the highest among the three siRNAs. Under hypoxic conditions, HeLa cells transfected with si-NC and si-p16#3 were subjected to 0 or 60 μM of A7G. (b) CCK-8 assay assessed the ability of HeLa cells to resist oxaliplatin and paclitaxel. (c) Colony formation assay assessed the proliferative ability of HeLa cells. (d) Western blotting assessment of the protein level of stemness-related proteins (SOX2, ALDH1, and Nanog). (e) Wound healing assay assessed the migration of HeLa cells. (f) Transwell assay assessed the invasive ability of HeLa cells. *n* = 3. Data are presented as the means  ±  SEM. *P* value was based on one-way analysis of variance. **P < 0.01, ***P < 0.001.

To determine whether the suppressive role of A7G in cervical cancer cells within the hypoxia environment relies on the activation of p16, we further explored the effect of A7G in p16 silencing HeLa cells. To prevent off-target effects, three siRNAs against p16 were designed for cell transfection. si-p16#3 was selected for further study as their knockdown efficiency was the highest among the three siRNAs (Figure 4a). Hela cells with the transfection of si-NC served as negative control. Subsequently, HeLa cells transfected with si-NC and si-p16 were exposed to 60 μM of A7G prior to the evaluation for proliferation, invasion, metastasis, and resistance to cell therapy. As expected, the resistance to chemotherapy, proliferation, stemness, migration, and invasion of HeLa cells with si-NC transfection was impaired by A7G (Figure 4b–f). However, in p16-silencing HeLa cells, such antitumor effects of A7G were not observed (Figure 4b–f), indicating that the inhibitory effect of A7G on the hypoxia-induced malignant phenotypes of cervical cancer cells is p16-dependent.

## 4 Discussion

As a distinctive characteristic of various types of solid cancer, hypoxia is associated with the acquirement of aggressive phenotypes of tumor cells, such as proliferation, resistance to chemotherapy, cancer stemness, metastasis, and immunosuppression [25]. DNA damage is considered the basic mode of action for a majority of classical anticancer drugs. Under hypoxic conditions, cancer cells undergo replication stress, leading to the activation of DNA damage and repair pathways [26,27]. Eventually, when present, hypoxia results in adverse prognosis in patients with solid malignancies, including cervical cancer [28]. Thus, exploring potential drugs for overcoming hypoxia-related chemo-resistance and metastasis is necessary. Several approaches for targeting hypoxic tumor cells have been proposed, including gene therapy, specific targeting of hypoxia-inducible factor-1, and hypoxia-activated prodrugs [29,30]. Cinnamaldehyde was identified to overcome hypoxia-induced oxaliplatin resistance, stemness, and epithelial–mesenchymal transition of colorectal cancer cells [31]. Based on previous reports, bortezomib could enhance the sensitivity of hypoxic cervical cancer cells to radiotherapy [32]. Moreover, plantamajoside was recently demonstrated to play a suppressive role in hypoxia-induced migration and invasion of human cervical cancer cells [33]. In the present study, stimulation under hypoxia induces HeLa cells to become more aggressive, which is consistent with the acquirement of aggressive phenotypes in multiple hypoxic cancer cell types [34,35].

A7G is a flavonoid that has been shown to have antitumor activity [36]. A7G can induce cell apoptosis and impair the migration of HeLa cells [23]. Nakazaki et al. reported that A7G could be a potential drug for the treatment of human promyelocytic leukemia by inhibiting the growth and granulocytic differentiation of HL-60 cells [22]. In our experiments, under hypoxic conditions, A7G not only suppressed proliferation, migration, invasion, and stemness but also strengthened the sensitivity to oxaliplatin and paclitaxel of HeLa cells. In addition, A7G influenced hypoxic HeLa cells in a dose-dependent manner.

As a well-known cyclin D kinase inhibitor encoded by CDKN2A, p16 is widely considered a tumor suppressor of which loss was shown to be related to poor outcome [37]. In general, p16 was found inactivated in a wide variety of malignant tumors [38,39]. However, p16 overexpression was observed in most cervical cancer and precancer cases because of the interaction of HPV oncogenes [40,41]. Hence, several reports considered p16 overexpression as a characteristic of neoplastic development of the cervical epithelium [42]. Nevertheless, whether p16 serves as an oncogene or tumor suppressor during the progression of cervical cancer remains unknown. A previous study revealed that p16 inhibited hypoxia-induced angiogenesis and metastasis in breast cancer [43]. Herein, we observed the involvement of p16 in hypoxia-induced malignant phenotypes of cervical cancer cells. A previous study has indicated a direct interaction between p16 and AE1 [44]. Moreover, AE1 protein could cause the cytoplasmic sequestration of p16, thereby promoting cancer progression [24]. Our data showed that hypoxia led to AE1 overexpression and p16 inactivation (nuclear protein levels were significantly reduced), which indicated that AE1 may be closely related to the inactivation of p16 in HeLa cells because of p16 sequestration in the cytoplasm. However, A7G treatment could reverse these changes. Molecular docking analysis indicated that A7G may interact with AE1 to enhance p16 activation. Therefore, the interaction of A7G with AE1 could save p16 from sequestration in the cytoplasm and allow more p16 to enter into the nucleus and become activated, thereby suppressing hypoxia-induced malignant phenotypes of HeLa cells. This finding also suggested that AE1/p16 may be a potential target for hypoxic tumor cells.

Finally, p16 silencing of HeLa cells was included in our study to verify whether the inhibitory effect of A7G on the hypoxia-induced malignant phenotypes of cervical cancer cells is dependent on p16. As expected, A7G had no significant effect on the resistance to chemotherapy, proliferation, stemness, migration, and invasion of p16-silencing HeLa cells. Thus, we hypothesized that A7G may impede the hypoxia-induced aggressive phenotype of HeLa cells by inhibiting AE1 protein expression.

It was the first to uncover the role of A7G in suppressing hypoxia-induced malignant phenotypes in cervical cancer cells and link these effects to the activation of AE1/p16 signal. Considering that cancer resistance could be addressed by accurately interfering with hypoxia to improve the therapeutic effect, the appropriate use of A7G could help tailor treatment for patients with cervical cancer in the future. However, some limitations are found in this study. For example, the detailed mechanism of A7G regulating AE1 expression must be further verified. In addition, animal experiments are lacking in this study. In the following experiments, we aimed to establish a tumor-bearing mouse model to determine whether A7G exerts antitumor effects *in vivo*.

## 5 Conclusion

The current study revealed that A7G repressed the proliferation, resistance to chemotherapy, stemness, migration, and invasion of cervical cancer cells under a hypoxic environment *in vitro*. Mechanistically, A7G could target the AE1, thereby reducing cytoplasmic sequestration of p16 and affecting the hypoxia-induced malignant phenotypes of cervical cancer cells. This result will provide an experimental basis for the application of A7G to the clinical treatment of cervical cancer.
